# A Custom-Made Newborn Screening Test for Wilson’s Disease in Puerto Rico

**DOI:** 10.7759/cureus.24446

**Published:** 2022-04-24

**Authors:** Raquel Vicario-Feliciano, Cristal I Hernández-Hernández, Ivonne C Camacho-Pastor, Juan C Martínez-Cruzado

**Affiliations:** 1 Department of Biology, University of Puerto Rico at Mayagüez, Mayagüez, PRI; 2 Internal Medicine, Perea Hospital, Bella Vista Hospital, Mayagüez, PRI

**Keywords:** taqman genotyping assay, european origin, preventive treatment, low-copper diet, the 1000 genomes project, cost-effectiveness, newborn screening, puerto rico, wilson’s disease

## Abstract

Background

Wilson’s disease (WD) is an autosomal recessive progressive, disabling, life-threatening disease. Although early diagnosis and treatment can halt disease progression and reverse disability, diagnosis is often challenging, with a mean diagnostic delay of approximately two years. At least 98% of WD-causing variants are in the *ATPase copper transporting beta* (*ATP7B*) gene. Identifying *ATP7B* mutations that cause WD in Puerto Rico will allow newborn screening for WD, as well as preventive, life-saving treatment.

Methodology

TaqMan genotyping assays were performed on 174 random volunteers in southwestern Puerto Rico and on three independent WD cases for rs367956522 and rs140708492, single-nucleotide polymorphisms (SNPs) composing a WD-causing haplotype. A polymerase chain reaction followed by Sanger DNA sequencing confirmed the case genotypes. Bioinformatics analyses were performed on *ATP7B* polymorphisms present in The 1000 Genomes Project (1KGP) database for Puerto Rico.

Results

rs367956522 is always inherited together with rs140708492 but not vice versa. The three independent WD cases were homozygous for both SNPs, but the evidence strongly suggested that rs367956522 is the pathogenic variant. The 1KGP database revealed the presence of only one other likely pathogenic *ATP7B* variant, rs191312027 (Gly869Arg). Together, both variants may be responsible for causing WD in one of every 14,156 Puerto Ricans. Both are likely of European origin.

Conclusions

Genotyping probes for both variants are readily commercially available. Thus, rapid, inexpensive newborn screening for rs367956522 and rs191312027 is strongly recommended. Although these two variants may account for all or the vast majority of WD cases in Puerto Rico, other *ATP7B *polymorphisms described or not described in this study might also be pathogenic.

## Introduction

Wilson’s disease (WD) is an autosomal recessive progressive disease caused by an excessive accumulation of copper in the body, especially in the liver and the brain. Although the mean age at which symptoms appear is 15.9 years, it varies widely [1]. Symptoms also show significant variation even between monozygotic twins [2] and may include liver malfunction, neurological and osseomuscular disorders, and the formation of the brownish-yellow Kayser-Fleischer ring around the corneal limbus [3]. WD is estimated to affect one per 7,000 individuals in the United Kingdom [4]. It is a disabling disease and is usually fatal by the age of 30 unless lifelong treatment is followed rigorously. Treatment includes a strict low-copper diet, drugs that bind and help remove copper, and zinc supplements that prevent copper absorption. Early diagnosis is critical for successful treatment. Followed by judicious decoppering, early diagnosis can halt disease progression and reverse disability, even to the point of reaching the asymptomatic stage for the rest of the patient’s life [3,5]. However, diagnosis is challenging and based on a combination of clinical features that do not always manifest. These include the Kayser-Fleischer ring, high levels of non-ceruloplasmin-bound copper in the blood, as well as high urinary copper excretion and hepatic copper content [6,7]. Consequently, the mean diagnostic delay is two years [8]. Newborn testing for rapid and early detection of WD combined with treatment long before symptoms appear can increase prognosis and quality of life substantially.

At least 98% of the WD cases in the United Kingdom are due to damaging mutations in the *ATPase copper transporting beta* (*ATP7B*) gene [4]. This gene encodes a P-type copper-transporting ATPase that activates ceruloplasmin, the main copper-transporting protein in the blood, by loading apoceruloplasmin with six copper molecules. *ATP7B* is also critical for the excretion of excess copper from the liver to the bile. Therefore, people with two malfunctioning copies of *ATP7B* suffer from defective copper metabolism and excretion, as well as excessive accumulation of copper in the body [9]. Although several transcripts have been predicted from this gene, only five have been curated. Our analyses focus on the curated transcripts which code for proteins 1465, 1387, 1381, 1354, and 1258 amino acids (aa) long.

The starting point of this study was a 2012 Mayo Clinic Laboratory Service Report for the sibling of a case of fulminant WD in Puerto Rico [10]. The laboratory sequenced all coding regions and exon/intron boundaries of the *ATP7B* gene and found the sibling to be heterozygous for a nucleotide substitution (G>A) in the splicing acceptor site of intron 11, a single nucleotide polymorphism (SNP) now known as rs367956522 and classified as pathogenic/likely pathogenic by NCBI/ClinVar [11]. It also found the SNP rs140708492, an intronic C>T transition that maintains a pyrimidine located within the pyrimidine tract that precedes the splicing acceptor site. rs140708492 is located 6 bp before the first nucleotide of exon 17 and has conflicting interpretations of pathogenicity in ClinVar.

We used TaqMan assays to genotype 174 random samples, mostly from southwestern Puerto Rico, for both SNPs. We further genotyped three additional cases of WD in southwestern Puerto Rico and found all cases and none of their family members to be homozygous for both SNPs. Finally, rs140708492 was found in the absence of rs367956522 but not vice versa. rs140708492 is a fairly common SNP in Puerto Rico, suggesting that rs367956522 is the major culprit of WD in southwestern Puerto Rico.

## Materials and methods

Recruitment and DNA extraction

Recruitment was undertaken in accordance with the approval of the Institutional Review Board (IRB) Committee of the University of Puerto Rico at Mayagüez (approval number: 00002053). Volunteers rinsed their mouths with 8 mL of distilled water, released the solution into a disposable plastic cup, and 2 mL of 100% ethanol was immediately added. Samples were stored at 4°C. For DNA extraction, samples were spun at 10,000 rpm for 10 minutes, and the pellets were resuspended in 200 µL of DNAzol and 10 µL of proteinase K at 20 mg/mL. After overnight incubation at room temperature, samples were spun at 14,000 rpm for 10 minutes, the supernatants were transferred to new microcentrifuge tubes, and 200 µL of 100% ethanol was added. The tubes were gently inverted several times, stored at -20°C overnight, and spun at 14,000 rpm for 15 minutes. DNA pellets were washed with 250 µL of 70% ethanol, dried, and resuspended in 200 µL of 100 mM Tris, pH 7.5, 0.1 mM Na_2_EDTA, pH 8.0.

DNA analysis

SNP genotyping was performed for rs367956522 and rs140708492 on a ViiA 7 Real-Time PCR System using Taqman SNP genotyping assays (Applied Biosystems, Waltham, MA, USA) according to the manufacturer’s instructions.

For DNA sequencing, amplicons were generated by polymerase chain reaction (PCR) using primers ATGAAAGAACAGGATCAATGTCAG and CTGTAATTGCGGGGTCTATAAATG to amplify a 537 bp fragment containing the rs367956522 site, and primers GGTGCTTACTTTTGTCTCTAACTG and ATCACTCGTAATCCTATTCCTTGG were used to amplify a 453 bp fragment containing the rs140708492 site. PCR conditions were 96°C for 1.5 minutes followed by 35 cycles of 96°C for 30 seconds, 51°C for 40 seconds, and 72°C for 60 seconds, plus a final step at 72°C for 5 minutes. Amplicons were purified using CleanSweep (Applied Biosystems, Waltham, MA, USA) as per the manufacturer’s instructions, vacuum dried, and sent for Sanger DNA sequencing to the Nevada Genomics Center.

Bioinformatics

Protein structural predictions were obtained by loading the full ATP7B protein sequence (NP_000044.2) onto the Phyre^2^ web portal for protein modeling, prediction, and analysis using default settings [12].

For the analysis of variants in *ATP7B* in Puerto Rico, the VCF file for chromosome 13, where *ATP7B* is located, was downloaded from The 1000 Genomes Project (1KGP), decompressed using 7-Zip, divided into 512 Mb files with GSplit, and the file containing the gene was opened and manipulated with Excel (Microsoft Office) using the headings in the first file. The region analyzed spanned the 2,706 polymorphisms that exist in the 1KGP database within the region starting with the polymorphism at the midpoint between the two reference SNPs (rs367956522 and rs140708492) and ending 100 kb upstream of the gene. The 2,705 polymorphisms downstream of the midpoint were added for a total of 5,411 polymorphisms, of which only 1,102 turned out to be polymorphic in any of the 112 individuals included in the analysis. The polymorphic sites generated sequence haplotypes for each of the 224 chromosomes that were converted into FASTA format using MEGA X [13], and then to RDF format using DnaSP [14]. The RDF file was loaded onto NETWORK5, and the median-joining network [15] was constructed using default settings.

Of the 1,102 polymorphisms found, only 12 were nonsynonymous. Predictions of the effect of these variants on protein function were obtained using Polyphen-2 [16] and SIFT [17].

## Results

Population frequency of rs367956522 and rs140708492

Table 1 shows the municipality distribution of the 174 random samples that were collected in southwestern Puerto Rico. Except for seven, all samples belonged to individuals residing in the municipalities between Añasco and Ponce. The minor allele of rs140708492 was found in its heterozygous form in five individuals (1.4% frequency). One of those five individuals was also heterozygous for rs367956522, which showed a frequency of 0.3%. The 1KGP sequenced the genomes of 104 Puerto Ricans, of which eight individuals were heterozygous for rs140708492. Again, one of those eight individuals was also heterozygous for rs367956522. Taking both sample sets together, rs140708492 appeared in 13 out of 278 individuals (2.3% frequency) while rs367956522 appeared in only two individuals (0.4% frequency), both heterozygous for rs140708492. As the chances of appearing randomly in two of the 13 individuals with the minor allele of rs140708492 but never in the 265 individuals without it are only 0.2% (χ^2^ = 41.08, p<< 10^-5^, df = 1), it is apparent that rs367956522 arose recently in a chromosome with the rs140708492 minor allele, and no recombination event has yet separated them.

**Table 1:** Municipality distribution and genotypes of random samples.

Municipality	n	# heterozygous for rs140708492 only	# heterozygous for rs140708492 and rs367956522
Añasco	2	1	---
Cabo Rojo	5	---	---
Comerío	1	---	---
Guánica	11	---	---
Guayanilla	9	---	---
Hormigueros	1	---	---
Lajas	13	---	---
Lares	1	---	---
Maricao	2	---	---
Maunabo	1	---	---
Mayagüez	7	---	---
Naranjito	1	1	---
Peñuelas	3	---	---
Ponce	4	---	---
Sabana Grande	16	2	---
San Germán	23	---	---
San Juan	2	---	---
Vega Baja	1	---	---
Yauco	71	---	1
Total	174	4	1

**Figure 1:**
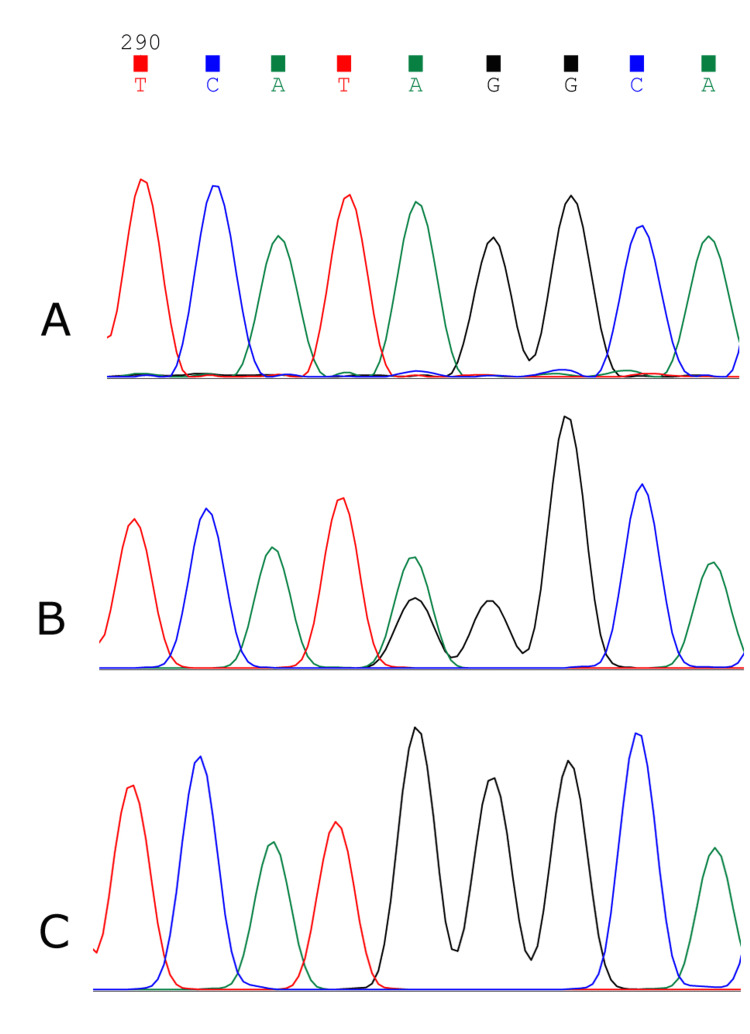
Chromatograms of the DNA region encompassing the splicing acceptor site of intron 11 in the ATP7B gene. (A)Control. The AG acceptor site is located at positions 294 and 295 in the sequence. (B) The heterozygous mother of the third WD patient. Both alleles (A (green) and G (black)) of rs367956522 appear at position 294.(C) The third WD patient. Homozygosity for the minor (G) allele at position 294 is observed. WD = Wilson’s disease

We also genotyped the sibling of a WD case previously reported [10] and 35 other members of the extended family, including a female WD patient. The patient was homozygous for the minor allele of both rs367956522 and rs140708492, and 10 of the remaining 35 members of the extended family were heterozygous for both SNPs. The rest were homozygous for the major allele of both SNPs. We further learned about two additional WD patients with no relation to this family. Both were women homozygous for the minor allele of both SNPs. Both parents of the second WD patient were heterozygous for both SNPs, as well as one of the patient’s two sisters. The mother of the third WD patient was also heterozygous for both SNPs. The father was unavailable for testing. The genotypes of all cases and their parents, when available, were confirmed by Sanger DNA sequencing (Figure 1).

Other polymorphisms possibly related to WD

Because the haplotype with the minor alleles of rs367956522 and rs140708492 was responsible for all three WD cases in independent families, we wondered if there was any other polymorphism in Puerto Rico capable of causing the disease. We sought for other potential WD-causing polymorphisms by searching the DNA sequences encompassing all exons and splice sites of *ATP7B* in the genomes of the 104 Puerto Ricans sequenced by the 1KGP. No splice site polymorphism was found other than rs367956522. However, we found 39 polymorphisms in the exons: three in the 5’ untranslated region, 23 in the coding region, and 13 in the 3’ untranslated region. Of those in the coding region, 12 were nonsynonymous and 11 synonymous. Table 2 shows the polymorphisms with their NCBI/ClinVar interpretations. Only two of them, rs191312027 (Gly869Arg) and rs189601972 (Gly1405Ser), have reports classifying them as pathogenic or likely pathogenic. However, Gly869Arg is a nonconservative replacement of a glycine located in the middle of a three-amino acid region whose structure, a hydrogen-bonded turn, was predicted with high confidence by Phyre^2^. It is part of the nucleotide-binding domain, which is located inside the E1-E2 ATPase domain (NCBI/Protein). Furthermore, 13 out of 14 ClinVar reports classify Gly869Arg as pathogenic or likely pathogenic. This polymorphism is predicted with high confidence by SIFT to affect protein function and is predicted to be probably damaging in all five isoforms by Polyphen-2. By contrast, Gly1405Ser is a conservative amino acid replacement occurring in a region predicted with rather low confidence to have a disordered structure outside of any protein domain. In addition, only one out of eight ClinVar reports classifies this polymorphism as pathogenic or likely pathogenic. It is predicted with low confidence by SIFT to affect protein function, and Polyphen-2 classifies it as probably damaging in only four isoforms. Hence, although a pathogenic effect cannot be discarded for Gly1405Ser, Gly869Arg is the only polymorphism other than rs367956522 highly likely to be pathogenic.

**Table 2:** Polymorphisms inside the five main ATP7B transcripts. ^∩^UTR = untranslated region ^*^According to build GRCh37 (hg19). Underlined positions indicate polymorphisms with a degree of likelihood for being pathogenic (see Discussion). ^∆^Identification number of the single nucleotide polymorphism in the National Center for Biotechnology Information database. ^†^CTCF = CCCTC-binding factor. Position of amino acid replacements refers to the largest protein. ^‡^Worldwide Minor Allele Frequency in The 1000 Genomes Project database. ^§^CIP = conflicting interpretations in pathogenicity; b = benign, lb = likely benign, us = uncertain significance, lp = likely pathogenic, p = pathogenic. ^#^1 to 5 refer to the ATP7B proteins from the longest to shortest. ^¥^SIFT = sorting intolerant from tolerant program. APT = affect protein function, low = low confidence prediction. ^||^Out of 208 Puerto Rican chromosome 13 copies. ^**^Polymorphism found only in Puerto Rico. ^ψ^Absent in the ATP7B protein 1381 aa long. ^Ω^Absent in the ATP7B protein 1258 aa long.

mRNA region^∩^	Position on Chr13^*^	dbSNP ID^∆^	Effect^†^	WMAF^‡^	ClinVar interpretation^§^	Polyphen-2 prediction^#^	SIFT prediction^¥ ^	Frequency in PR^||^
5’ UTR	52585591	rs148013251	CTCF binding site	0.37	CIP: 5b, 1lb, 1us	---	---	77
5’ UTR	52585548	rs2277448	CTCF binding site	0.48	Benign	---	---	80
5’ UTR	52585504	rs184427928	Unknown	<0.01	Not reported	---	---	1
Protein coding	52549258	rs184868522	Met33Thr	<0.01	Uncertain significance	1. Benign 2, 3, 4. Possibly damaging 5. Probably damaging	1, 2, 4. APT (low) 3, 5. Tolerate (low)	2
Protein coding	52548320	rs201200863^**^	Pro346Ser^ψ^	<0.01	Not reported	Benign	Tolerated (low)	1
Protein coding	52548158	rs199807461	Thr400Ala	<0.01	CIP: 1lb, 2us	1, 2, 5. Benign 3, 4. Possibly damaging	Tolerated	1
Protein coding	52548140	rs1801243	Ser406Ala	0.38	Benign	Benign	Tolerated	75
Protein coding	52544809	rs150018860	Synonymous	<0.01	Likely benign	---	---	1
Protein coding	52544805	rs1801244	Val456Leu	0.38	Benign	Benign	Tolerated (low)	78
Protein coding	52542667	rs145798966	Synonymous	<0.01	CIP: 3b, 5lb, 2us	---	---	1
Protein coding	52524488	rs1061472	Lys832Arg	0.50	Benign	1, 2, 3, 4. Benign 5.Possibly damaging	Tolerated	99
Protein coding	52524268	rs191312027	Gly869Arg	<0.01	CIP: 8p, 5lp, 1us	Probably damaging	APT	1
Protein coding	52523808	rs732774	Arg952Lys^Ω^	0.47	Benign	Benign	Tolerated	98
Protein coding	52520507	rs1801246	Synonymous	0.02	Benign	---	---	7
Protein coding	52520501	rs200656411^**^	Synonymous	<0.01	Likely benign	---	---	1
Protein coding	52520471	rs1801247	Synonymous	0.04	Benign	---	---	10
Protein coding	52520435	rs1801248	Synonymous	0.01	CIP: 8b, 1lb	---	---	11
Protein coding	52516568	rs59120265	Synonymous	0.01	Benign	---	---	3
Protein coding	52516529	rs373081328	Synonymous	<0.01	CIP: 1b, 4lb, 1us	---	---	1
Protein coding	52515354	rs1801249	Val1140Ala	0.46	Benign	Benign	Tolerated (low)	96
Protein coding	52513266	rs7334118	His1207Arg	0.03	CIP: 7b, 2lb, 1us	1, 3, 4, 5. Benign 2. Probably damaging	Tolerated	11
Protein coding	52509077	rs189601972^**^	Gly1405Ser	<0.01	CIP: 1lp, 7us	1, 2, 3, 5. Probably damaging 4. Possibly damaging	APT (low)	1
Protein coding	52508989	rs60986317	Thr1434Met	0.02	CIP: 1b, 3lb, 8us	1, 4. Possibly damaging 2, 3, 5. Probably damaging	Tolerated (low)	1
Protein coding	52508988	rs116091486	Synonymous	0.01	Benign/Likely benign	---	---	3
Protein coding	52508979	rs73202048	Synonymous	<0.01	Benign/Likely benign	---	---	4
Protein coding	52508895	rs199859839^**^	Synonymous	<0.01	Likely benign	---	---	1
3’ UTR	52508877	rs73498144	CTCF binding site	<0.01	Likely benign	---	---	2
3’ UTR	52508823	rs566159129^**^	CTCF binding site	<0.01	Not reported	---	---	1
3’ UTR	52508739	rs563069856	Unknown	<0.01	Not reported	---	---	1
3’ UTR	52508702	rs115420019	Unknown	0.03	1b, 1us	---	---	7
3’ UTR	52507883	rs79747858	Unknown	0.01	Likely benign	---	---	1
3’ UTR	52507856	rs533209080^**^	Unknown	<0.01	Uncertain significance	---	---	1
3’ UTR	52507816	rs564499990	Unknown	<0.01	Uncertain significance	---	---	1
3’ UTR	52507720	rs1051332	Unknown	0.31	Benign	---	---	70
3’ UTR	52507710	rs79490882	Unknown	0.04	Benign	---	---	7
3’ UTR	52507370	rs191892694^**^	Unknown	<0.01	Not reported	---	---	1
3’ UTR	52507175	rs77770386	Unknown	0.02	Benign	---	---	5
3’ UTR	52507148	rs9535794	Unknown	0.02	Likely benign	---	---	9
3’ UTR	52507110	rs928169	Unknown	0.48	Benign	---	---	101

The origin of WD-associated variants in Puerto Rico

In an effort to learn more about the origin of rs367956522, we constructed a median-joining network of all 104 Puerto Rican haplotypes in the 1KGP. Because the minor allele of rs367956522 is always found in the company of the minor allele of rs140708492, we suspected that rs367956522 arose in a chromosome with the minor allele of rs140708492, and thus decided to include the haplotypes of all non-Puerto Rican individuals with the minor allele of rs140708492 in the 1KGP. These were eight individuals: three from British in England and Scotland (GBR), two from Utah with Northern and Western European ancestry (CEU), and one each from Toscani in Italy (TSI), Mexican ancestry in Los Angeles, California (MXL), and Bengali in Bangladesh (BEB). In addition, eight Puerto Ricans had the minor allele of rs140708492, including one with the minor allele of rs367956522.

**Figure 2:**
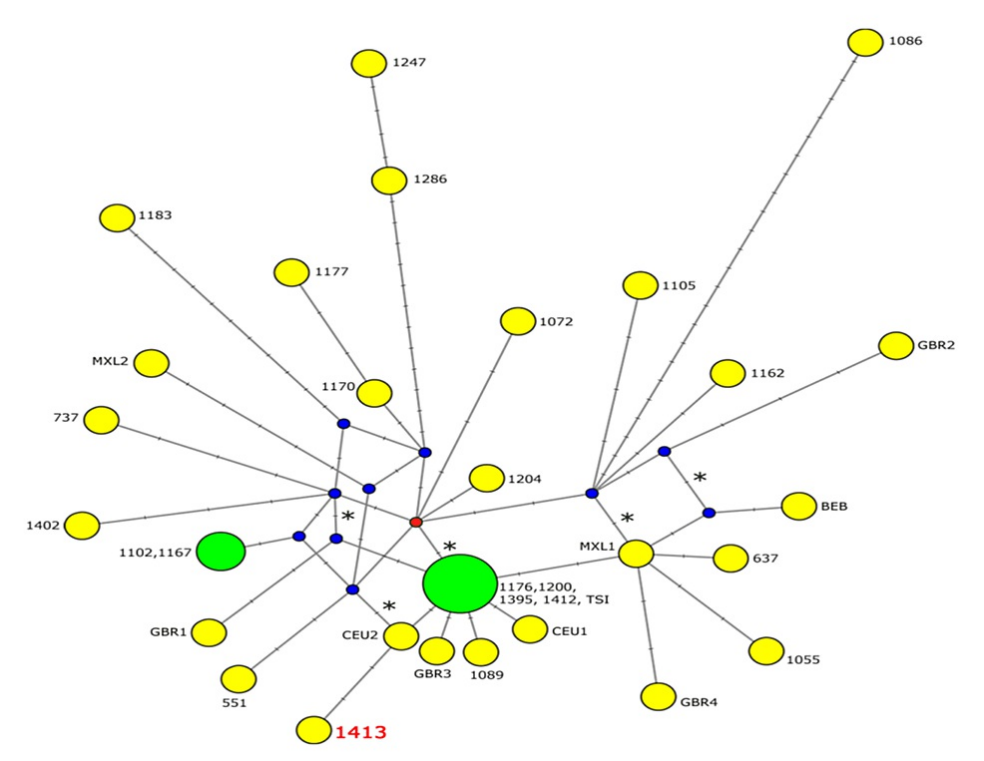
Median-joining haplotype subnetwork of all haplotypes whose most recent hypothetical ancestors differ from the haplotype shared by five individuals by not more than four mutations. Haplotypes are represented by circles with size proportional to the number of chromosomes they represent. Haplotypes represented by one chromosome are shown in yellow, and those represented by more than one chromosome in green. The 1KGP ID of each sample to which each chromosome belongs is shown next to its corresponding circle: three or four-digit numbers represent the ID of Puerto Rican samples (e.g., 1402 = HG01402), while non-Puerto Rican chromosomes are identified by letters (e.g., GBR# = chromosomes from British in England and Scotland, see text). The haplotype with the pathological mutation rs367956522 (1413) is highlighted in red and a bigger, bold font. Hypothetical ancestor haplotypes are represented by small blue circles except for the root ancestor haplotype, which is highlighted as a small red circle. Crosshatches denote mutations distinguishing haplotypes from one another. Crosshatches representing rs140708492 are highlighted by asterisks. Only one mutation distinguishes the haplotype shared by five chromosomes from the root, rs140708492, which forms part of four of the seven reticulations.

The resulting network consisted of six major subnetworks, one of which included all 16 chromosomes with the minor allele of rs140708492. Five of the chromosomes shared the same haplotype, which differed from the root of the subnetwork only by rs140708492. Figure 2 shows the subnetwork which we defined as including all haplotypes whose most recent hypothetical ancestor differs by not more than four variants from the haplotype shared by five chromosomes. The subnetwork contains seven reticulations, and rs140708492 participates in four of them, suggesting it is a fairly old SNP. The five chromosomes sharing the haplotype are four from Puerto Rico and one from Italy. In consistency with its old age, the shared haplotype has already given rise to four other haplotypes (CEU1, CEU2, GBR3, and HG0189 from Puerto Rico). The haplotype with the pathological variant of rs367956522 (HG01413), shown in red and bold, differs from CEU2 by only two mutations. One of them is rs367956522 and the other is rs145144325, a 1 bp deletion within a stretch of 14 thymines within intron 1.

We also searched for the Puerto Rican haplotype holding the Gly869Arg variant within the network, HG01095. It was found isolated in a subnetwork different from that containing rs140708492 (not shown). It was close only to another Puerto Rican haplotype (HG00743) and differed from it by three mutations: rs191312027 (Gly869Arg), a mutation unique to it in the whole 1KGP database, and rs145144325 (not shown). Because it is a 1 bp deletion within a stretch of 14 thymines, rs145144325 is expected to be hypermutable, and its presence in both subnetworks analyzed, as well as its high worldwide frequency (0.24), is consistent with that expectation. However, it was still informative. The 1KGP database only has four samples in addition to HG01095 with Gly869Arg. We searched for all four, of which two are British and two Chinese haplotypes, and both British haplotypes had the rs145144325 1 bp deletion whereas both Chinese haplotypes did not. This data together with the finding of the 1KGP Admixture working group [18], which concluded that the region encompassing the *ATP7B* gene was of European ancestry for both copies of chromosomes 13 in HG01095, strongly suggest that the Gly869Arg version of Puerto Rico is of European origin.

## Discussion

*ATP7B* diversity in Puerto Rico and feasibility of neonatal screening for WD

Newborn screenings are meant to detect genetic conditions for which efficient treatments exist and whose application early in life is critical for their success. WD is a disabling and potentially fatal disease whose symptoms can be reversed with rigorous and timely treatment. However, diagnosis is highly challenging and often achieved only after symptoms have progressed to the point of no return. Although only one gene, *ATP7B*, appears to be responsible for the totality of cases, more than 600 pathogenic variants in *ATP7B *have been identified [9]. Hence, for most populations, making reliable detection is only possible through costly methods such as exome sequencing.

However, Puerto Rico is a special case. Its population history is marked by founder events and bottleneck effects [19-21] that reduce diversity. Consequently, we may expect a very limited number of mutations responsible for WD. Under that scenario, designing a newborn screening test for WD in Puerto Rico may be feasible.

We have now tested WD cases in three independent families that all showed homozygosity for the same deleterious mutation, rs367956522. In addition, in a population survey, we found only two other SNPs with reports classifying them as pathogenic or likely pathogenic in ClinVar. They have very low worldwide frequencies (<0.01), as expected from pathogenic variants. rs191312027 (Gly869Arg) is the most likely pathogenic of the two. It has been reported in compound heterozygous cases of WD in the Canary Islands [22], Spain [23], and repeatedly in China [24,25]. ClinVar interpretations for Gly869Arg range from “pathogenic” to “uncertain significance,” and in WilsonGen, a clinically annotated genomic variant resource specific for WD [26], all 12 entries classify Gly869Arg as pathogenic or likely pathogenic following the guidelines of the American College of Medical Genetics and the Association of Molecular Pathologists. WilsonGen further reports Gly869Arg cases in Italy, Sardinia, and in an Anglo Saxon. Polyphen-2 prediction scores span from 0.998 to 1 for this SNP in the five isoforms of *ATP7B*. In Polyphen-2, “1” represents the maximum probability of being damaging. Gly869Arg represents the replacement of a glycine residue, which is small, polar but of neutral charge, for arginine, a big amino acid of positive charge. The replacement occurs in exon 11 which is a part of the E1-E2 ATPase domain. The second SNP, rs189601972 (Gly1405Ser), was found only once in the 1KGP, and it was in Puerto Rico. To our knowledge, there is no mention of this SNP in the medical literature, perhaps because of its limited geographic distribution and very low population frequency. It has only one entry in WilsonGen, which claims insufficient evidence to interpret its potential pathogenicity. The glycine at position 1405 is evolutionarily conserved in other mammal superorders represented by the dog (Laurasiatheria) and the elephant (Afrotheria), as well as in the platypus (Subclass Prototheria), the chicken (Class Aves), Xenopus (Class Amphibia), and the zebrafish (Class Actinopterygii), presumably because it plays an important role. Polyphen-2 predicts this amino acid replacement to be probably damaging in four of the five main ATP7B proteins, and possibly damaging for the other. In addition, SIFT predicts this SNP to affect protein function in all isoforms, albeit with low confidence. On the other hand, the location of Gly1405Ser on exon 21 argues against its pathogenicity because none of the 116 ATP7B mutations reported as associated to WD cases in a population study in the United Kingdom [4], and none of the 20 similarly reported in China [25] were located on this exon. In addition, none of the *ATP7B* variants with likely pathological significance in the UniProt database, and none of the 396 entries of nonsynonymous variants classified as pathogenic in WilsonGen localize to exon 21. In summary, although computational models and the evolutionary conservation of this variant suggest it may be pathogenic, there is a lack of experimental evidence supporting its pathogenicity.

All other coding region variants found lacked reports in ClinVar classifying them as pathogenic or likely pathogenic (Table 2). All had reports classifying them as benign or likely benign except for Met33Thr (rs184868522), which was only classified as of uncertain significance. Met33Thr has two entries in WilsonGen, with both classifying it as likely benign. Predictions of the effect of this variant on protein function are largely contradictory: Polyphen-2 scores this variant as possibly damaging in three isoforms and probably damaging in another, but SIFT predicts, always with low confidence, that the variant is tolerated when present in two isoforms, including the isoform where it is probably damaging according to Polyphen-2, and affecting protein function when present in the remaining three isoforms. In the protein sequence, amino acid site 33 precedes any domain and any variant of likely pathological significance in the UniProt database, and its methionine is conserved only in mammals and birds. We conclude that Met33Thr (rs184868522) is not likely to be pathogenic.

Polymorphisms in the untranslated regions (UTRs) have not been studied as thoroughly as those in the coding region. However, these polymorphisms can play important roles in the regulation of transcription and translation of mRNAs as well as their transportation, localization, and stability. Of the 16 polymorphisms in the UTRs, four have not been reported in ClinVar, two have been interpreted as of uncertain significance, and all others have reports classifying them as benign or likely benign, but none as pathogenic or likely pathogenic. Entries in WilsonGen are largely consistent with those in ClinVar.

In summary, there is only one variant described in Puerto Rico in addition to rs367956522 with the certainty of being pathogenic. Although rs367956522 appears to be the major WD variant in southwestern Puerto Rico, Gly869Arg (rs191312027) may be the cause of many WD cases in other regions on the island. Considering the measured frequencies of both variants, 2/556 for rs367956522 and 1/208 for rs191312027, we estimate that they together cause WD in one of every 14,156 Puerto Ricans.

There are two additional missense variants with a chance of being pathogenic, especially Gly1405Ser (rs189601972). Other variants with a slight chance of being pathogenic are two variants in the 3’ UTR classified as of uncertain significance, and four variants in both UTRs that have not been reported in ClinVar. All these variants have worldwide frequencies of less than 0.01 (Table 2).

In conclusion, newborn screening in Puerto Rico for WD likely requires tests for only rs367956522 and Gly869Arg (rs191312027). Tests should be performed in additional WD cases to confirm the absence in Puerto Rico of other WD-causing variants, including other polymorphisms described in this study that have a slight chance of being pathogenic. Specifically, the tests should first inquire about the possible presence of rs367956522 and Gly869Arg (rs191312027), and if they are not present in their homozygous or compound heterozygous forms, then try Gly1405Ser (rs189601972), Met33Thr (rs184868522), and the six polymorphisms located in the UTRs that have not been reported or that have been interpreted as of uncertain significance. *ATP7B* exome sequencing should be undertaken only after all these polymorphisms are shown not to be in their homozygous or compound heterozygous forms in a WD case.

Origin of WD-causing variants in Puerto Rico

The minor allele of rs367956522 is always inherited together with the minor allele of rs140708492, showing that they are inherited in *cis*. However, it is unlikely that the minor allele of rs140708492 plays a major role in WD. It is a transition that maintains a pyrimidine within the pyrimidine tract that precedes the splicing acceptor site of intron 16. Having tested three WD cases, and all being homozygous for rs367956522 when rs140708492 has a frequency 6.5 times higher provides strong statistical evidence for discarding rs140708492 as pathogenic (see Results). Furthermore, rs140708492 is not a low-frequency variant among British, where its frequency is 1.6%, and yet has never been reported in its homozygous form or as a compound heterozygous in any WD case in the absence of rs367956522. By contrast, despite its far lower worldwide frequency, rs367956522 has been reported in both homozygous and compound heterozygous forms in WD cases. rs367956522 eliminates the splicing acceptor site of intron 11. Inclusion of intron 11 sequences in the mRNA results in premature stop codons that eliminate the last 555 aa of the protein, including the complete phosphorylation domain [9]. Interestingly, only when in its homozygous form an alternative splicing site is used that maintains the reading frame, with the effect of producing a protein that differs from wild-type only by a 13 aa insertion at the beginning of exon 12. However, the resulting protein does not resolve pathogenicity [27].

As both polymorphisms are only 10,599 bp apart, the minor allele of rs367956522 is always inherited together with the minor allele of rs140708492. Thus, we combined all samples carrying rs140708492 in the 1KGP dataset with the Puerto Rican samples in our median-joining network analysis in an effort to understand the origin of rs367956522 in Puerto Rico. Minor alleles have generally their highest frequency in their population of origin [28]. rs140708492 has its highest frequency in Great Britain, and rs367956522 has been found only in Great Britain and the Americas. Thus, it is reasonable to propose that the pathogenic haplotype made it to Puerto Rico from Great Britain, either directly or indirectly.

The median-joining network suggests that rs140708492 has arisen only once, and is an old polymorphism, giving rise to several haplotypes, some of them present in four of the seven reticulations in the subnetwork. Its origin in Great Britain is supported by its higher frequency there and its presence in populations with British introgression such as Bangladesh (BEB) and the United States (CEU, Figure 2). On the other hand, one of its haplotypes is shared by four Puerto Ricans and a Toscani, suggesting that the origin of haplotypes containing rs140708492 but not rs367956522 in several Puerto Ricans could be in Italy. However, the same haplotype is likely present in Great Britain as it has given rise to other haplotypes present in Great Britain and the United States. One of them, CEU2, is the haplotype closest to the Puerto Rican pathogenic haplotype (HG01413). It differs from HG01413 only by the pathogenic mutation (rs367956522) and a likely hypermutable 1 bp deletion within a 14 bp thymine homopolymer. Thus, the Puerto Rican pathogenic haplotype is very closely related to haplotypes likely of British origin that are now independently shown to have migrated to the Americas.

Curiously, the origin of the other confirmed WD pathogenic variant in Puerto Rico, rs191312027 (Gly869Arg), is also likely to be Great Britain. The variant shows up in both Great Britain and China in the 1KGP database, but only in Great Britain is it linked to the 1 bp deletion within a 14 bp thymine homopolymer, as it is in Puerto Rico. In addition, the chromosomal segment holding rs191312027 (Gly869Arg) in Puerto Rico was determined to be of European ancestry through local ancestry analysis. Thus, WD may be expected to afflict mostly Puerto Ricans of higher European ancestry.

Limitations of this study

This study has been limited by its sampling. Samples from the northern and eastern regions, which are the most populated regions of Puerto Rico, were virtually absent from our sampling. This limitation was partly addressed by incorporating the analysis of the 1KGP sample set of Puerto Rico. However, although the 1KGP sample set is more equally distributed across Puerto Rico, its European ancestry is higher than the average for the Puerto Rican population. Thus, our sample set is not entirely representative of the Puerto Rican population. The most probable effect of this limitation on our conclusions is that we may have overestimated the number of Puerto Ricans with WD caused by homozygosity or compound heterozygosity of rs191312027 and rs367956522 (one in 14,156 Puerto Ricans). Another possible effect is that we may have missed other pathogenic variants in Puerto Rico, especially any originating outside of Europe.

## Conclusions

WD is usually fatal unless timely and strict lifelong treatment is implemented. Early diagnosis is critical for effective treatment, but diagnosis is often challenging, with the average delay between the appearance of symptoms and diagnosis being two years. Fortunately, 98% of cases can be traced to mutations in only one gene. The Mendelian mode of inheritance of the disease allows for newborn screening and subsequent intervention prior to symptom onset.

By testing three cases in southwestern Puerto Rico, surveying 174 healthy subjects in the region, and further analyzing the database of the 1KGP for the population of Puerto Rico, we conclude that all or almost all cases in this population can be attributed to only two polymorphisms, both likely of European origin. Other polymorphisms with a slight degree of likelihood to be pathogenic are also identified. We propose further cases be screened for the two polymorphisms, and predict that the patients will be homozygous for either of them or compound heterozygous. If our predictions turn out to be correct, we strongly recommend that rapid and cost-effective newborn screening for these two polymorphisms be implemented immediately in Puerto Rico.
